# Unraveling human transferrin-tryptamine interactions: a computational and biophysical approach to Alzheimer’s disease therapeutics

**DOI:** 10.3389/fphar.2025.1540736

**Published:** 2025-03-19

**Authors:** Mohammed Alrouji, Mohammed S. Alshammari, Taghreed A. Majrashi, Azna Zuberi, Moyad Shahwan, Akhtar Atiya, Anas Shamsi

**Affiliations:** ^1^ Department of Medical Laboratories, College of Applied Medical Sciences, Shaqra University, Shaqra, Saudi Arabia; ^2^ Department of Clinical Laboratory Sciences, College of Applied Medical Sciences, Shaqra University, Shaqra, Saudi Arabia; ^3^ Department of Pharmacognosy, College of Pharmacy, King Khalid University, Abha, Saudi Arabia; ^4^ Division of Reproductive Science in Medicine, Department of Obstetrics and Gynecology, Feinberg School of Medicine, Northwestern University, Chicago, IL, United States; ^5^ Center for Medical and Bio-Allied Health Sciences Research, Ajman University, Ajman, United Arab Emirates; ^6^ Department of Basic Medical Sciences, College of Applied Medical Sciences, King Khalid University (KKU), Muhayil, Asir, Saudi Arabia

**Keywords:** Alzheimer’s disease, human transferrin, molecular docking, molecular dynamics simulation, fluorescence spectroscopy

## Abstract

Neurodegeneration is a progressive loss of neurons that leads to affected cognitive and motor functions and is characterized by neurodegenerative disorders (NDs). Human transferrin (Htf) is a blood plasma glycoprotein that binds to iron and regulates the free iron in biological fluids. Free iron is a potent neurotoxin associated with the generation of Reactive oxygen species (ROS) and is ultimately linked to oxidative stress and neuronal damage. Thus, targeting iron homeostasis is an attractive strategy for the management of NDs, viz. Alzheimer's disease (AD). Tryptamine (Trp) is a naturally occurring monoamine, that has demonstrated promising roles in AD therapeutics. The present study aims to delineate the binding mechanism of Trp with Htf employing computational and spectroscopic approaches. Molecular docking ascertained the vital residues governing the Htf-Trp complex formation. Further, Molecular dynamic (MD) studies ascertained the structural dynamics and stability of the complex, implying that the binding of Trp causes minimal structural alterations in Htf, suggestive of the stability of the complex. The results from fluorescence spectroscopy demonstrated the binding of Trp with Htf with a binding constant (*K*) of 0.48 × 10^6^ M^−1^, validating the *in silico* observations. This study provides a platform to understand the binding mechanism that may lead to novel therapeutic approaches targeting AD.

## 1 Introduction

Neurodegenerative disorders (NDs) describe a group of illnesses marked by a gradual and progressive loss of nervous system structure and function, primarily affecting neurons. These disorders typically lead to a steady decline in cognitive abilities, motor skills, and other neurological functions (Szigeti, 2020). NDs impact millions of people worldwide. Although aging is the leading risk factor for developing these non-communicable diseases, recent studies reveal that genetics and environmental factors also play significant roles. An individual’s immediate environment influences the extent and rate of neurodegeneration (Jain et al., 2012; Jain and Chen-Plotkin, 2018), even while specific genes responsible for NDs are expressed (within an individual) (Liu et al., 2022). Recent research indicates that a neurodegenerative condition is linked with several other disorders (Brouwer-DudokdeWit et al., 2002; Esch et al., 2002; Allan and Rothwell, 2003; Liu et al., 2017). As a result, NDs can be extremely dangerous or even fatal in some cases; this all depends on the kind and stage of the disease. The Alzheimer’s Association states that Alzheimer’s disease (AD) causes damage to brain regions essential for basic bodily functions like walking and swallowing. This condition is fatal, and research shows that most people diagnosed with Alzheimer’s after age 65 typically live only a few more years (Association, 2019).

Despite the increasing availability of supportive and therapeutic options, AD remains a leading cause of dementia, affecting millions of people worldwide. This number is expected to rise significantly in the near future (Poirier et al., 1993; Olajide and Sarker, 2020).

Neurodegeneration is characterised by sudden neuronal loss and abnormal synaptic connections with can lead to imbalances in the neurotransmitters and therefore onset of memory loss. Selective memory loss is one of the early signs of onset of AD (Szigeti, 2020; Szigeti, 2020). Neuroinflammation is one of the major factor associated with AD progression (Heppner et al., 2015; Hesse et al., 2016; Fu et al., 2018). Out of various factors influencing the onset and progression of AD, tau pathology, Aβ aggregation, and iron homeostasis have gained wide attention. Iron (Fe) is an essential and most abundant element essential for the growth and development of humans (Saini et al., 2024). In human brain, Fe is associated with various important functions such as axon myelination, neuronal division and synthesis of neurotransmitters. However, in the ageing brain, Fe accumulates, leading to neurodegeneration and neuroinflammation (Mezzanotte et al., 2024).

The inflammatory process, through interactions with iron-regulatory proteins (IRPs), affects Fe homeostasis by producing ROS and reactive nitrogen species (RNS) (Xue et al., 2022). Although the precise onset of neuroinflammation in NDs is unknown, the activation of astrocytes and microglia triggers a strong inflammatory response (Adamu et al., 2024).

Human transferrin (Htf) is one of the most prevalent serum proteins in plasma and is involved in the transportation of both internal and external materials (Sarzehi and Chamani, 2010). With 679 amino acids and two metal-binding sites that have about 30% iron saturation, this single-chain glycoprotein connects to the Fe-transferrin receptor and the cell’s endosomal compartment to play a critical role in iron transport (Hadzhieva et al., 2014; Moghaddam et al., 2014; Sharifi-Rad et al., 2021; Behjati Hosseini et al., 2022; Khan et al., 2023). Fe deposition in neurons is caused by pro-inflammatory cytokines changing Fe-related proteins that preserve Fe homeostasis. In AD, early neuroinflammation causes Fe loading in specific brain areas.

Tryptamine (Trp) is an alkaloid monoamine that occurs naturally and is generated from the amino acid tryptophan. The alkaloid is used as a psychedelics drug which are psychoactive substances that alter perception, mood, and cognitive processes (Raj et al., 2023). Trp shows a wide range of biological activities, such as inhibition of factors associated with AD, including Aβ aggregation and monoamine-oxidase activity. It also shows anti-oxidant effects and neuroprotective effects (Singh and Kumar, 2023). Supplementary Figure S1 shows the structure of Trp. In the present work, binding of Trp with the protein Htf was analysed in detail using *in silico* and *in vitro* experiments. By analyzing the relationship between Htf and Trp, this study investigates the possible therapeutic value of focusing on Fe homeostasis in AD.

## 2 Materials and methods

### 2.1 Material

Human transferrin (Htf) and Trp were purchased from Sigma-Aldrich Co. (St. Louis, MO, United States). The dialyzed protein was filtered with a syringe filter and used for further studies. The stock prepared was 5 mg/mL, which was diluted for fluorescence binding studies in sodium phosphate (PBS) buffer accordingly. The inner filter effect was considered during spectroscopic investigations.

### 2.2 *In Silico* toxico-kinetic predictions

The toxicokinetic characteristics of Trp were ascertained through web-based resources, which included pkCSM and SwissADME (Daina et al., 2017; Pires et al., 2015), as mentioned in our previous publication (Shamsi et al., 2024). The chemical structures of Trp in smiling format were used as inputs to run the predictions using their default parameters.

### 2.3 Molecular docking

Molecular docking was used to assess the molecular interactions and binding affinities between Htf and Trp. The Protein Data Bank provided the three-dimensional structure of transferrin (PDB ID: 3V83), while the PubChem database provided the Trp structure (Compound CID: 1,150), which was then processed using InstaDock. During the docking process, a blind search space method was used to exhaustively examine potential binding sites for the ligands by spanning the complete protein structure. The structures were viewed using Discovery Studio to investigate the binding conformation and interactions between Trp and Htf.

### 2.4 MD simulations

Molecular dynamics (MD) simulations in drug discovery and molecular biology have grown significantly in past decades. The MD study shows proteins behaviour in complete atomic detail (Hollingsworth and Dror, 2018). A 200 ns simulation was run to assess the structural and conformational stability of Htf with Trp. GROMACS v5.5.1 was used to carry on the simulations (Van Der Spoel et al., 2005). The detailed MD protocol used is described in our previous publications (Anwar et al., 2022; Shamsi et al., 2022). Energy minimization was carried out for all the systems using the steepest descent algorithm and neutralization of the charges. The system temperature was increased from 0 to 300K. The final MD run of 200 ns was carried out on all the systems. Quality check metrics were used to verify the resultant simulations for Htf and its ligand complex.

### 2.5 Principal component analysis (PCA) and free energy landscape (FEL)

PCA is an effective and commonly used tool to analyze the functional motion modes of protein structures. PCA was performed to discover the conformational projection of Htf and Htf-Trp complex using the *gmx_covar*, *gmx_anaeig* tools of the Gromacs. It was based on the calculation and diagonalization of the covariance matrix as cited (Altis et al., 2008). PCA analysis was carried out on the MD simulation equilibrated trajectories. The analysis was carried out to understand the structural alterations in the protein structure in its native form and after binding of the ligand. The structural dynamics of apo-Htf and Htf-Trp were further studied by the FEL models as described in our previous publications (Shamsi et al., 2021; Adnan et al., 2023; Anwar et al., 2023).

### 2.6 Fluorescence spectroscopy

A fluorescence binding assay was carried out on a spectrofluorometer (Shimadzu RF-6000). 5 μM was dissolved in PBS buffer. Trp was dissolved in DMSO to make a stock solution of 1 mM and stored in a dark eppendorf to avoid photodegradation. Htf was excited at 280 nm and the fluorescence spectra were recorded from 300–400 nm. The resulting fluorescence data were plotted as intensity versus Trp concentration, and binding curves were generated. The data was fitted in the Modified Stern–Volmer equation to find the binding constant (*K*).

## 3 Results and discussions

### 3.1 Toxico-kinetics prediction

Toxicokinetic properties are the traits and mechanisms that control how toxins behave within the body of an organism. The characteristics include the compound’s toxicological effects and absorption, distribution, metabolism, and elimination (ADME). Using the tools SwissADME, pkCSM, and ProTox, the toxicokinetic properties were predicted *in silico*. Table 1 summarizes the projected ADMET features. Trp weighs 160.22 g/mol, has a tiny molecular structure, a high lipophilic area, and relatively little polar surface area. The value of Log Po/w (iLOGP) is 1.54. This number represents the compound’s expected partition coefficient as determined by the iLOGP method. It represents the compound’s hydrophobicity and propensity to partition between octanol, a non-polar solvent, and water, a polar solvent. According to the consensus value of 1.53, Trp has moderate lipophilicity. Compoundswith Log Po/w values more than 1 are generally considered lipophilic. Trp is confirmed to be lipophilic because its consensus Log Po/w value is greater than 1, which suggests that it has a greater affinity for lipid environments than aqueous environments. Utilizing SwissADME, pkCSM, and ProTox tools, *in silico* predictions were utilized to predict the toxicokinetic characteristics of Trp. Table 1 provides an overview of the generated ADMET properties.

**TABLE 1:** ADMET properties obtained from SwissADME, pkCSM, and ProTox.

ADMET parameter	Value
Molecular Weight (g/mol)	160.22 g/mol
Number of hydrogen bond acceptors	1
Number of hydrogen bond donors	2
Topological Polar Surface Area (Å^2^)	41.81 Å^2^
Water solubility (Log S)	‒1.70
Octanol/water partition coefficient (log P_o/w_)	2.46
GI absorption (% Absorbed)	High
Skin permeability (logKp)	−6.78 cm/s
Fraction unbound (Fu)	0.42
BBB permeability (log BB)	Yes
P-Glycoprotein Substrate	Non-Substrate
Half-Life of Drug	Half-Life < 3hs
Total clearance (log mL/min/kg)	8.9
Predicted LD_50_ (mg/kg)	940 mg/kg

Lipophilic molecules tend to congregate in lipid-rich areas of the body because of their strong affinity for nonpolar substances. Compared to hydrophilic chemicals, Trp is more easily absorbed due to its lipophilic character, making it easier to pass through biological barriers and penetrate cell membranes. According to the ESOL and Ali models, Trp is extremely highly soluble in water. It is plausible to conclude that Trp is water soluble and has a high probability of rapidly absorbing and penetrating through the blood-brain barrier (BBB) in light of these strong solubility predictions. Nevertheless, the substance has a relatively poor skin permeability (−6.78 cm/s). It is reported that the oral LD50 for Trp is 940 mg/kg. Higher LD50 values are often associated with lower acute toxicity and reduced hazardousness in chemical safety and risk assessment. ProTox tool predictions indicate that Trp belongs to toxicity category 4 and is classified as having no harmful effects. Even at moderate dosages, the chemical does not appear to have any immunogenic, carcinogenic, or mutagenic properties.

### 3.2 Molecular docking

**FIGURE 1:**
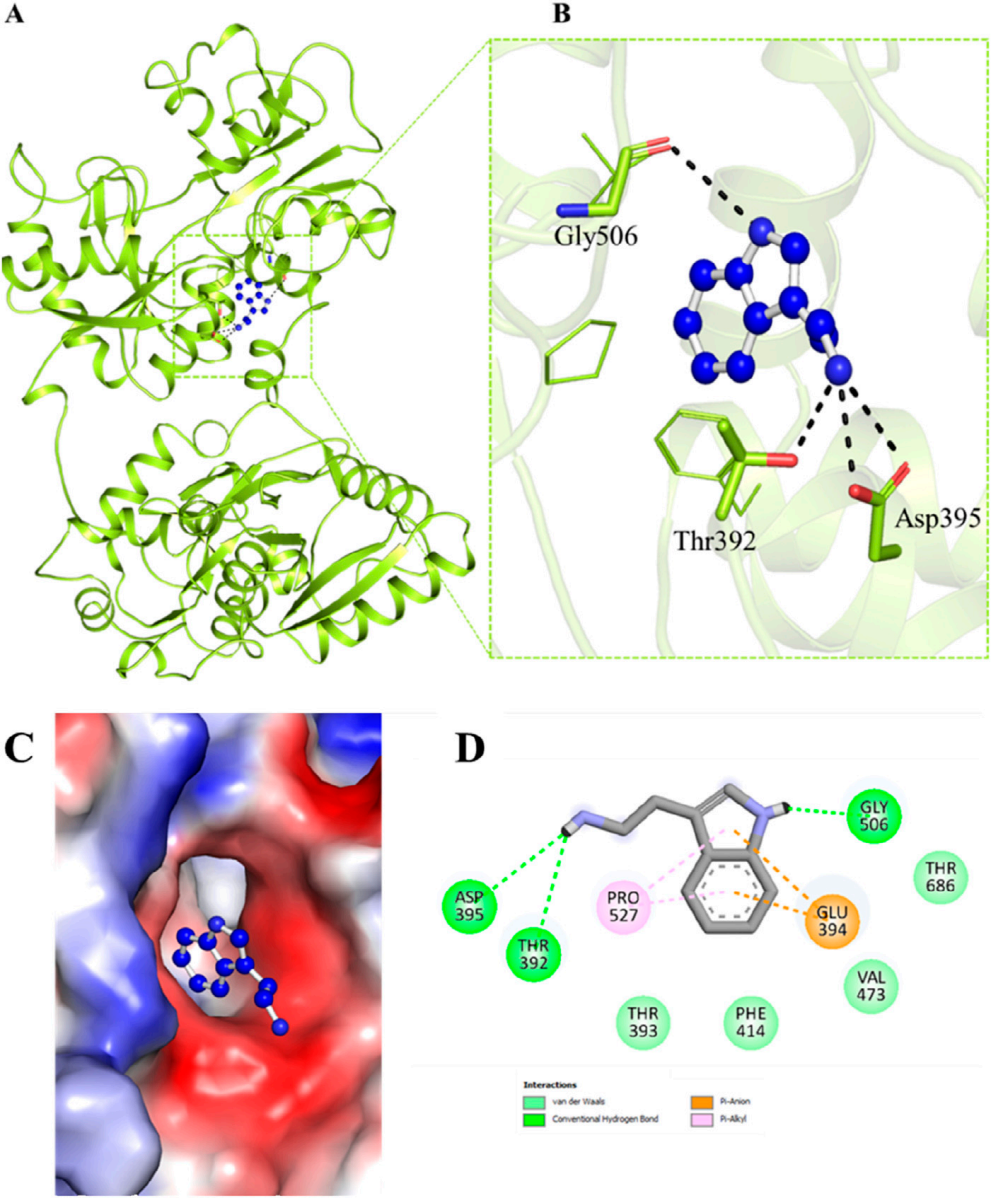
**(A, B)** Cartoon representation showing the docked Trp interacting with Htf. The residues involved in hydrogen bonding are visible in a zoomed-in view of the binding pocket. **(C)** Surface view of Trp in the binding pocket of Htf. **(D)** 2-D structural representation of Htf residues interacting with Trp.

Molecular docking is a routinely employed method to have an insight into the binding of ligands with a protein and to analyse which residues are playing a key role in the interaction (Alkhathami et al., 2025). Here, structure-based docking was carried out to investigate the binding prototype of Trp within the Htf binding site. To investigate potential interactions between Trp and the significant residues of Htf, interaction analysis of the docked conformers of Trp was performed. The best pose was chosen based on the interaction with key residues of the binding pocket. Trp formed various interactions with several crucial binding pocket residues (Figure 1A, B) while occupying a deep position in the binding pocket, as seen in Figure 1C Htf is depicted as a surface in Figure 1C, with Trp depicted as ball and stick model holding a place in the binding pocket. The 2-D structural model of Htf residues interacting with Trp is shown in Figure 1D.

Trp interacts with Htf with various interactions such as Asp395, Gly506, Thr686, Val473, Thr393, and Phe414 form van der Waals interactions, Thr392 is associated with hydrogen bond formation. Furthermore, Glu394 and Pro527 interact with the Trp aromatic ring through a pi-anion and pi-alkyl interaction, respectively. As shown in the Figure 1D, Trp attaches to Htf via hydrogen bonds, van der Waals forces, and pi interactions. The Trp’s aromatic ring is critical in pi-related interactions with Pro527 and Glu394. Trp binding to the Htf protein is stabilised by the combined effects of these several interactions.

### 3.3 MD simulations

Computational methods such as molecular dynamics (MD) simulations are widely employed to study the dynamics and structural alterations in proteins and protein-ligand complexes. An effective method for researching biomolecular interactions over predetermined time intervals is to use MD simulations. This work focused on Htf and Htf-Trp docked complex and ran a thorough 200 ns MD simulation. Our main goals were to investigate conformational alterations, evaluate stability, and clarify the fundamental mechanisms controlling the interaction between Trp and Htf. We used the simulated trajectory’s root mean square deviation (RMSD) to assess structural changes.

**FIGURE 2:**
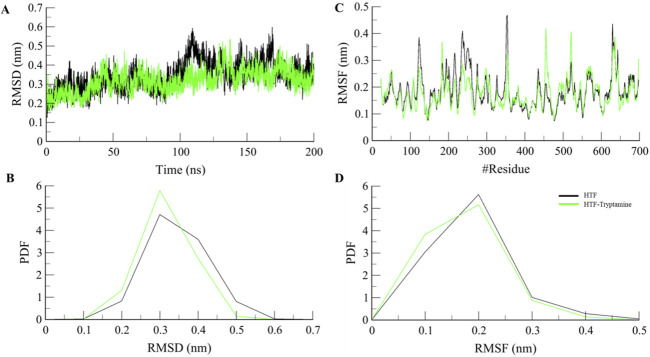
Structural dynamics **(A)** RMSD, **(B)** PDF of RMSD, **(C)** RMSF, and **(D)** PDF of RMSF plot of Htf and Htf-Trp complex.

The binding of Trp to Htf shows the formation of a stable complex as shown in Figures 2A, B. In a 200 ns simulation, we observed that the apo-protein showed mean RMSD value of 0.34 nm, while the protein-ligand complex’s mean RMSD value of 0.32 nm indicated stable complex formation with minimum fluctuations in the values. We computed and plotted the simulated systems’ root-mean-square fluctuation (RMSF) to investigate local structural alterations and residue flexibility. Interestingly, there was a consistent trend in the overall average RMSF of Htf and Htf-Trp complex. Both Htf and Htf-Trp have comparable RMSF patterns, as shown in Figures 2C, D.

**FIGURE 3:**
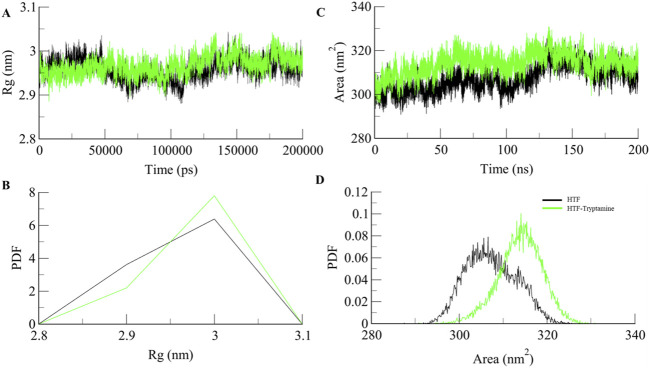
Structural compactness **(A)** R_g_, **(B)** PDF of R_g_, **(C)** SASA, and **(D)** PDF of SASA plot.

The radius of gyration (*R*_*g*_) measures a protein’s overall size and how its mass is distributed around its center. An increase in *Rg* suggests the protein adopts a more expanded and less compact shape. *Rg* value showed an increase of 1 nm upon ligand binding Figures 3A, B, which indicates a significant expansion of the protein upon ligand binding, likely due to the protein adopting a more open or extended form. This expansion can make active sites or functional regions more accessible, potentially enhancing the protein’s interactions with other molecules or substrates. However, while such structural changes can improve functionality, they can also impact stability. A higher *R*_*g*_ may expose hydrophobic regions to the solvent or disrupt stabilizing interactions, potentially decreasing overall stability. Nonetheless, the increased flexibility and dynamic range could benefit the protein’s function, especially if it undergoes significant conformational changes or needs to accommodate various substrates or partners.

The solvent-accessible surface area (SASA) measures how much of a protein’s surface is exposed to the surrounding solvent. An increase in SASA upon ligand binding shows that the protein’s surface is accessible due to the binding of the ligand, as shown in (Figures 3C, D). SASA values show a significant increase from 307.29 nm^2^ for apo-Htf to 313.9 nm^2^ for the Htf-Trp complex. These changes can increase the protein’s flexibility and expose additional surface areas, enhancing its ability to interact with other molecules or substrates (Durham et al., 2009).

### 3.4 Stabilization of Htf-Trp complex and secondary structure predictions

**FIGURE 4:**
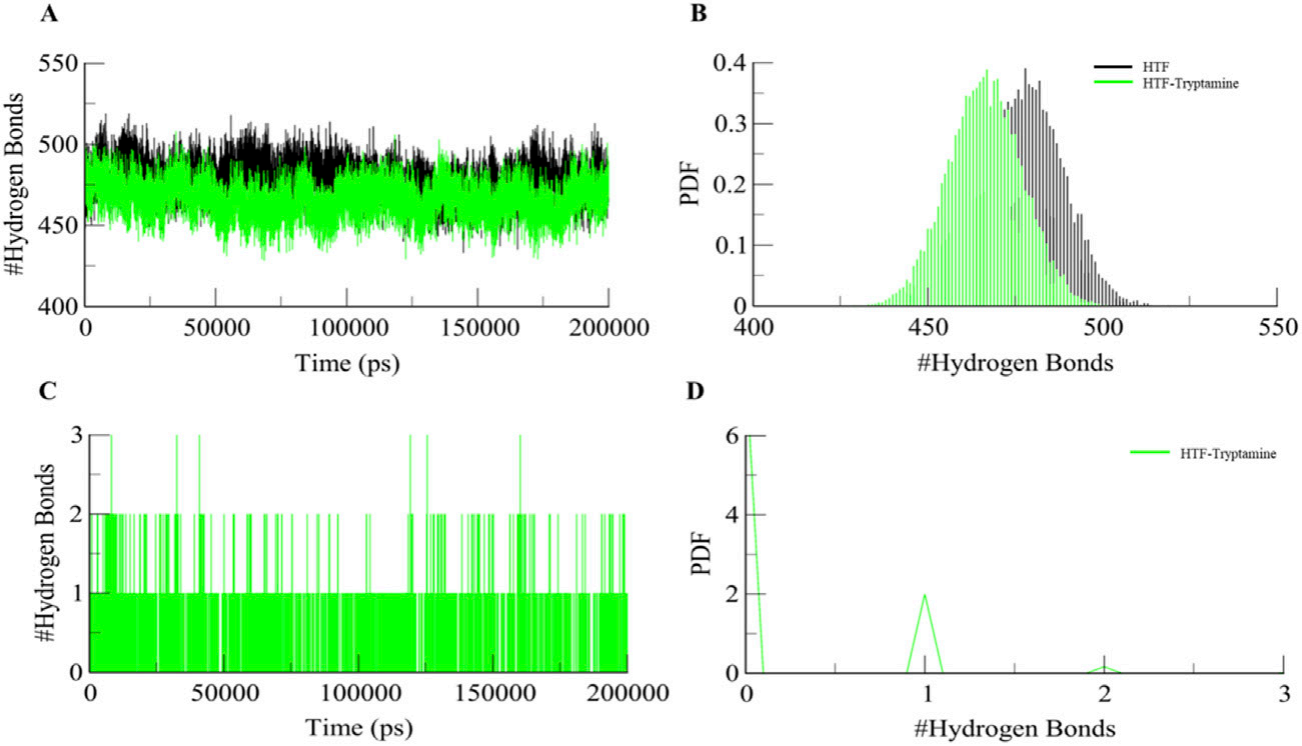
**(A)** Dynamics of Intramolecular hydrogen bonds in free Htf (Black) and Htf-Trp (Green). **(B)** PDF of Intramolecular hydrogen bonds. **(C)** Dynamics of intermolecular H-bonds in Htf-Trp and **(D)** PDF of intermolecular hydrogen bonds.

Intramolecular hydrogen bonding is an important factor in maintaining the integrity of protein structure. Examining these hydrogen bonds provides important information on how stable the polar interactions are inside protein complexes. To evaluate the stability of the protein both in complex with the ligand and on its own during the simulation, we performed a comprehensive study of intramolecular hydrogen bonding (Figures 4A, B). This investigation demonstrates how the creation of hydrogen bonds in Htf varies with and without Trp. The simulation showed that the intramolecular hydrogen bonds did not significantly change, suggesting that Htfs structural integrity is maintained even after Trp binding. A reduction in intramolecular hydrogen bonds within Htf upon ligand binding indicates significant structural changes in the protein. This decrease may signal increased flexibility and the conformational adjustments needed for effective ligand interaction (Menéndez et al., 2016; Yunta, 2017).

We also performed intermolecular hydrogen bonding analysis during the simulation between Htf and the bound ligand. Notably, the bound Trp ligand remained within the binding pocket throughout the simulation, indicating minimal deviation from its initial docking position (Figures 4C, D). This suggests that Trp forms stable interactions with Htf. The analysis of intermolecular hydrogen bonding revealed persistent hydrogen bond interactions between Trp and key residues of Htf. The number of hydrogen bonds fluctuated within a narrow range, reinforcing the hypothesis that Trp binding does not significantly alter the protein’s conformational dynamics but rather stabilizes the complex.

**FIGURE 5:**
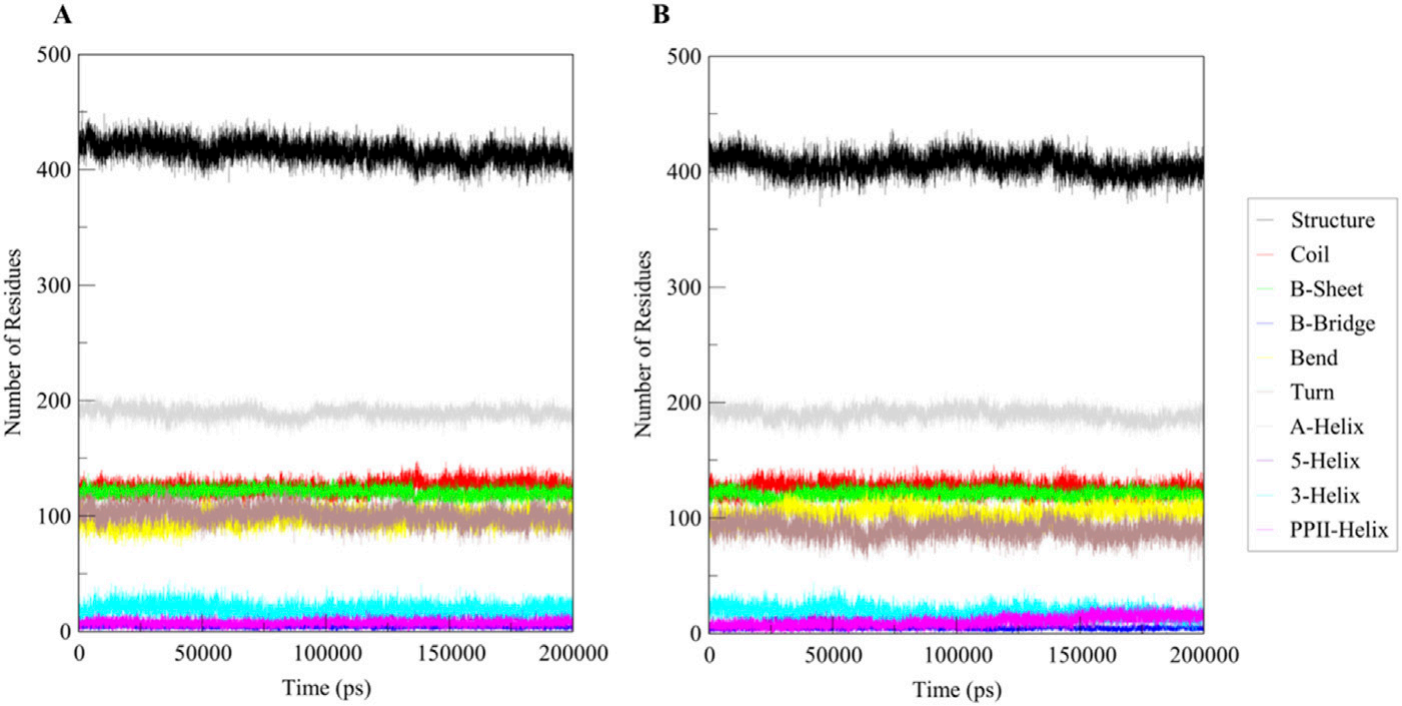
Secondary structural dynamics in **(A)** Free Htf and **(B)** Htf bound with Trp.

The secondary structure of Htf remains highly stable upon ligand binding, preserving its α-helices, β-sheets, and other structural elements. This indicates a robust protein framework. Ligand binding may cause minor localized changes near the interaction site, but these are not significant enough to alter the overall structure, as shown in Figure 5. Htf’s function likely depends on minor, precise adjustments at the binding site or tertiary structure level rather than large-scale rearrangements. The binding site appears to be specifically pre-organized to accommodate the ligand efficiently, allowing for binding with minimal structural disruption.

### 3.5 Free energy landscape analysis and principal component analysis

**FIGURE 6:**
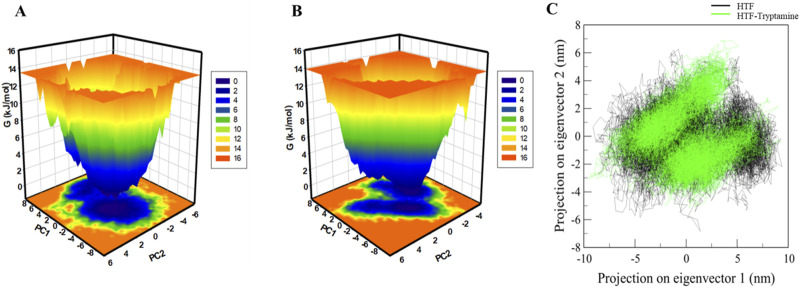
Free energy landscape: **(A)** contour map for the Htf. **(B)** Htf-Trp. **(C)** Principal component analysis of Htf and Htf-Trp complex.

FEL analysis provides crucial insights into the stability and conformational preferences of protein-ligand complexes. We examined the FELs produced by the simulated trajectories to better understand the systems’ folding landscape. The FELs for Htf and Htf-Trp complex are displayed in Figure 6. The FEL analysis can distinguish proteins and protein-ligand complexes between their kinetic and thermodynamic states. Discrete rainbow representations were used to create FELs, which were then used to evaluate the overall stability and folding mechanism of Htf before and after Trp binding (Figure 6A). On the other hand, the addition of Trp causes a noticeable global minimum centered in a single basin (Figure 6B). The formation of a deep global energy minimum upon tryptamine binding suggests that the transferrin-tryptamine complex adopts a stable conformational state, reinforcing the docking and MD simulation findings. Interestingly, this binding event does not result in any substantial structural rearrangements. This implies that Trp binding modifies the energy landscape of Htf and adds novel conformational states. Analysing these FELs offers important insights into the stability of the conformation stability of Htf and Htf-Trp complex.

According to (Stein et al., 2006), PCA offers important insights into conformational landscapes and protein stability. This work investigated the conformational dynamics of Htf and Htf-Trp complex from the simulated trajectory using PCA. The conformational dynamics of these systems along eigenvectors 1 and 2 are shown in Figure 6C. According to the PCA plot, Htf investigates a smaller variety of phase spaces when Trp is present. Htf’s motion did not exhibit any major conformational changes or significant overarching transitions after Trp binding, suggesting that Trp binding does not cause substantial conformational changes in Htf.

### 3.6 Fluorescence binding assay

**FIGURE 7:**
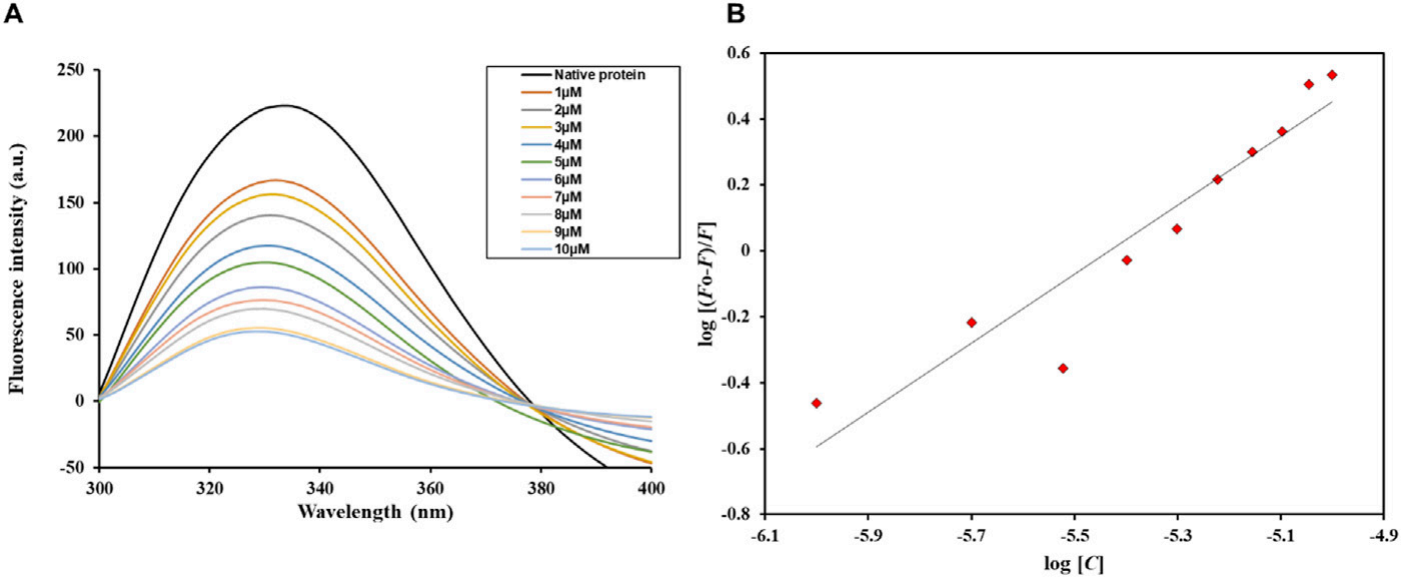
**(A)** Fluorescence emission spectra of free Htf and Htf in the presence of varying Trp concentrations (1–10 µM). **(B)** Modified Stern–Volmer spectra of the Htf-Trp complex.

According to the graph illustrating the fluorescence quenching assay results (Figure 7), Htf’s fluorescence intensity gradually decreases when the ligand Trp concentration rises from 1 μM to 10 μM. With each Trp addition, the fluorescence intensity of the original protein (shown by the black curve) systematically decreased from its peak. This quenching result points to a direct binding relationship between Trp and Htf, which lowers fluorescence emission by changing the immediate environment around the fluorophore or causing conformational changes. The slow decline in peak height, indicative of the quenching pattern, is consistent with a binding affinity (*K*) of 0.48 × 10^6^ M^−1^. The fluorescence emission spectra’s constant shape across a range of Trp doses indicates binding occurs at a single location or in a comparable protein microenvironment. Furthermore, the lack of a shift in the emission wavelength suggests that the binding interaction lowers the quantum yield and has no discernible effect on the Htf’s overall structural configuration or its fluorophore surroundings. Since no spectrum shifts are seen, this consistent decrease in fluorescence intensity across all concentrations points to a conventional static quenching mechanism rather than a dynamic quenching (Fossum et al., 2023).

## 4 Conclusion

The study provides comprehensive insights into the role of Trp in modulating the function of Htf, a key glycoprotein involved in Fe transport, and its implications in NDs like AD. NDs, particularly AD, are marked by the progressive loss of neuron structure and function, with neuroinflammation and iron dysregulation being pivotal factors in disease progression. Trp was evaluated for its binding affinity with Htf through molecular docking, revealing significant interactions within the protein’s binding pocket. The current study suggests that Trp might influence Htf’s role in maintaining iron homeostasis, a critical factor in AD pathology.

The study’s findings also highlight the potential therapeutic implications of Trp in AD treatment. By modulating Htf activity and, thus, iron homeostasis, Trp could be a promising candidate for reducing neuroinflammation and oxidative stress, which are key contributors to neurodegeneration in AD. In conclusion, the research underscores the significance of iron homeostasis in AD and the potential of Trp in AD therapeutics in the context of iron homeostasis offering a new avenue for therapeutic intervention in NDs.

## Data Availability

The original contributions presented in the study are included in the article/Supplementary Material, further inquiries can be directed to the corresponding authors.
