# Supplementary material for: Unraveling human transferrin-tryptamine interactions: a computational and biophysical approach to Alzheimer’s disease therapeutics
Source: Front Pharmacol. 2025 Mar 19;16:1540736. doi: 10.3389/fphar.2025.1540736 (PMC11962429; doi:10.3389/fphar.2025.1540736)
Supplement: Supplementary file 1 [file Image1.pdf]

# Unraveling Human Transferrin-Tryptamine Interactions: A Computational and Biophysical Approach to Alzheimer's Disease Therapeutics

Mohammed Alrouji[1], Mohammed S. Alshammari[2], Taghreed A. Majrashi[3], Azna Zuberi[4], Moyad Shahwan[5], Akhtar Atiya[6#]Anas Shamsi[5*]

[1]*Department of Medical Laboratories, College of Applied Medical Sciences, Shaqra University, Shaqra 11961, Saudi Arabia. Email:* malrouji@su.edu.sa
[2]*Department of Clinical Laboratory Sciences, College of Applied Medical Sciences, Shaqra University, Shaqra, 11961, Saudi Arabia. Email:* M.Alshammari@su.edu.sa
[3]*Department of Pharmacognosy, College of Pharmacy, King Khalid University, Abha, Kingdom of Saudi Arabia. Email:* tamajrashi@kku.edu.sa
[5]*Division of Reproductive Science in Medicine, Department of Obstetrics & Gynecology, Feinberg School of Medicine, Northwestern University, Chicago, Illinois, USA. Email: azna.zuberi@northwestern.edu*
[5]*Center for Medical and Bio-Allied Health Sciences Research, Ajman University, UAE. Email:* anas.shamsi18@gmail.com
[6]*Department of Basic Medical Sciences, College of Applied Medical Sciences, King Khalid University (KKU), Muhayil, Asir, Saudi Arabia. Email:* atkhan@kku.edu.sa

***#Co Corresponding***
***Akhtar Atiya Khan***
*College of Applied Medical Sciences*
*King Khalid University (KKU),*
*Muhayil, Asir, Saudi Arabia*

***Corresponding Author***
***Anas Shamsi, PhD, MRSB**
Centre of Medical and Bio-allied Health Sciences Research
Ajman University
United Arab Emirates.
Email: anas.shamsi18@gmail.com

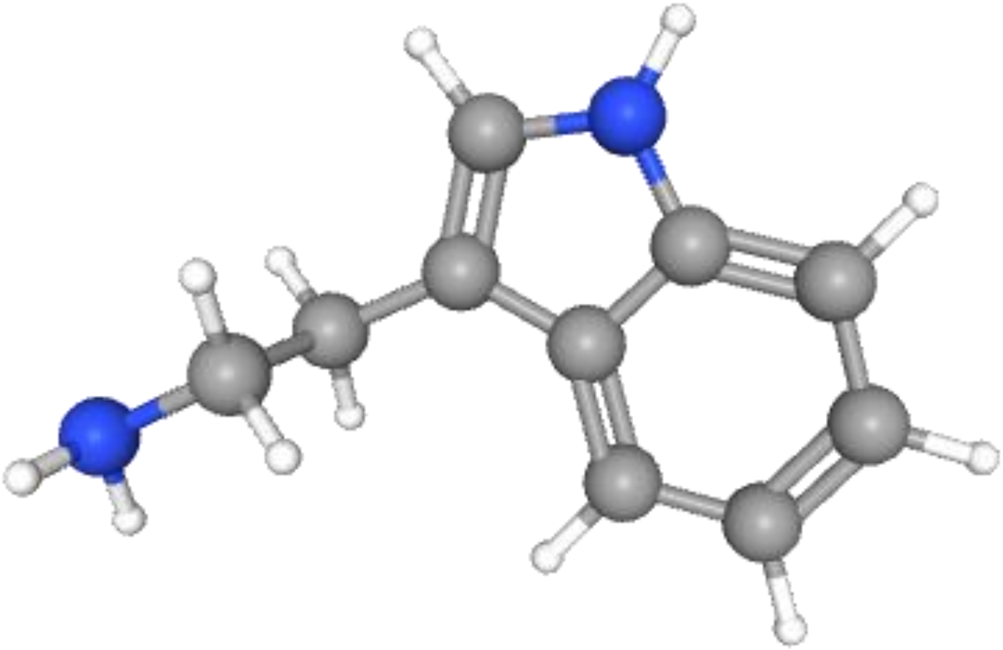

*Figure S1: Structure of Trp.*
